# Improved Separation in Horizontal Protein SDS-PAGE with Double-Deck Flat Electrodes and a Field Inversion Gel Electrophoresis Module

**DOI:** 10.3390/mps6060106

**Published:** 2023-11-03

**Authors:** Dong Woo Lim, Tae-Sung Yoon, Kyung Ho Han, Saba Sajjad, Heung-Seon Shin, Sunghyun Kang

**Affiliations:** 1 T-MAC Co., Ltd., Yuseong-gu, Daejeon 34141, Republic of Korea; limdw@tmacbio.com (D.W.L.); hsshin@tmacbio.com (H.-S.S.); 2 Critical Diseases Diagnostics Convergence Research Center, Korea Research Institute of Bioscience and Biotechnology (KRIBB), Yuseong-gu, Daejeon 34141, Republic of Korea; yoonts@kribb.re.kr (T.-S.Y.); bint.e.sajjad6@gmail.com (S.S.); 3 Department of Biological Sciences and Biotechnology, Hannam University, Yuseong-gu, Daejeon 34054, Republic of Korea; kyungho1@hnu.kr

**Keywords:** horizontal PAGE, electrode, FIGE

## Abstract

The horizontal flatbed electrophoresis method is employed to separate protein samples, providing greater flexibility for various electrophoretic applications and easier sample loading compared to its vertical counterpart. In the currently available equipment setup, cathode and anode electrodes are positioned on top of a gel at each end. Since an electric field enters the gel from the top, its strength gradually weakens from the top to the bottom of the gel. When examining the interior of gels following electrophoretic separation, the uneven electric field causes the protein bands to lie down forward in the direction of migration, leading to an increase in bandwidth. This issue has remained unaddressed for several decades. To address this problem, new clamp-shaped and double-deck electrodes were developed to apply an electric field simultaneously from both the top and bottom of the gel. Both of these new electrodes facilitated the formation of perpendicular protein band shapes and enhanced resolution at a comparable level. Due to their ease of use, double-deck electrodes are recommended. By combining these new electrodes with the field inversion gel electrophoresis (FIGE) technique, the protein bands could be focused and aligned nearly vertically, resulting in the highest level of electrophoretic resolution. Our electrodes are compatible with polyacrylamide gels of varying sizes, buffer systems, and sample well formats. They can be easily manufactured and seamlessly integrated into existing laboratory instruments for practical use.

## 1. Introduction

Sodium dodecyl sulfate (SDS)-polyacrylamide gel electrophoresis (PAGE) is a well-established and reliable method for separating proteins based on their molecular weight [1]. It can be easily performed using affordable laboratory equipment. With its unique capability to visualize proteins in a gel, SDS-PAGE provides insights into protein size and purity, which may not be as readily accessible using alternative methods. SDS-PAGE has been widely used in conjunction with Western blotting or mass spectrometry analysis techniques, further enhancing its utility [2,3].

This technique has only changed marginally over the past five decades. There are two types of apparatus for protein SDS-PAGE: vertical and horizontal electrophoresis systems [4]. They both follow the same electrophoretic principles, but the main difference is in the installation of gels. In the vertical PAGE system, a gel is held within two glass plates and mounted between two buffer chambers. The only electric path between the two buffer chambers is through the gel. In the horizontal PAGE system, an unsupported gel is placed onto the cooling plate, and a pair of electrodes connect with the gel to provide an electric field. The electrolytes are provided using buffer-soaked wicks without using buffer chambers.

The vertical PAGE system is preferred for routine protein separations. Cooling the gels from both sides enables thicker gel accommodation, thereby enhancing the protein loading capacity and facilitating easier gel handling after electrophoresis. Furthermore, the vertical system allows for the simultaneous operation of multiple gels. In contrast, the horizontal PAGE system excels in its versatility for various electrophoretic applications, such as isoelectric focusing (IEF). A comparative study demonstrated that the horizontal SDS-PAGE system exhibited higher resolution and sharper protein spots than the vertical system in two-dimensional polyacrylamide gel electrophoresis (2D-PAGE) [5].

Another advantage of the horizontal electrophoresis system is the ability to load samples directly onto the top of a gel, enabling the separation of a large number of samples in a single gel. Exploiting this feature, Ciaccio et al. developed the microwestern array (MWA) [6]. In their study, 672 samples and size markers were electrophoresed together on a large-sized gel. Subsequently, the separated proteins were transferred to a blotting membrane, and distinct antibodies were applied to different sections of the membrane. While the MWA inherited certain benefits and limitations from regular Western blotting, its poor electrophoretic resolution stood out as a significant drawback. This limitation could be attributed to the significantly shorter electrophoretic separation distance in the MWA. This drawback inspired our study to explore improved methods for achieving better separation resolution in horizontal electrophoresis.

For separating a wide range of proteins in a single lane, the protein bands must be more focused for higher resolutions. Laemmli’s discontinuous SDS-PAGE system has continuously been improved in many ways. Using a concentration gradient gel instead of a single concentration gel has extensively improved the resolution of complex protein mixtures [7], although, even with an extensive gradient range, smaller proteins could not be resolved well with a standard buffer system. Adding urea to the buffer improved the separation of small peptides but hampered proper molecular weight determination [8]. An alternative was to optimize the ionic constituents in buffers from Laemmli’s original system [9,10,11]. Efficient removal of Joule heating was also crucial to preventing band broadening [12]. Field inversion gel electrophoresis (FIGE) was one of the pulsed field gel electrophoresis techniques originally invented to separate large DNA molecules [13]. In FIGE, the polarity of an electric field is periodically reversed with a longer time or a higher voltage gradient in one direction; therefore, a size-dependent resolution can be achieved. FIGE is the easiest to perform with minimal equipment requirements, yet successfully maximizes efficiency and selectivity for large DNA molecules. FIGE was successfully implemented with protein PAGE to reduce band diffusion and to increase the protein concentration in a band [14].

All commercially available horizontal PAGE systems used electrodes disposed on top of a gel at both ends. In such a configuration, an electric field was stronger and caused faster migration of proteins in the top portion of the gel. Hence, the protein bands were broadened and not clearly separated from the others. In this study, we developed new electrode designs which could rectify this flaw. The FIGE technique was also tested together with double-deck electrodes to achieve the best resolution for protein electrophoresis.

## 2. Materials and Methods

### 2.1. Reagents

The 30% acrylamide (29:1), TEMED, and Precision Plus Protein™ Dual Color Standards were purchased from Bio-Rad (Hercules, CA, USA). The tris-HCl, ammonium persulfate, and sodium dodecyl sulfate were purchased from Sigma (St. Louis, MO, USA). All reagents used were of analytical grade.

### 2.2. Fabrication of SDS-Polyacrylamide Gels

The mixture for the SDS-polyacrylamide gels was prepared by combining an acrylamide and bis-acrylamide solution (10% T, 3.3% C), 375 mM Tris-HCl (pH 8.8), 0.1% SDS, 0.1% APS, and 0.04% TEMED in distilled water. Subsequently, the mixture was poured into a laboratory-made gel caster (Supplementary Figure S1) and left for polymerization. Careful handling was necessary while placing the gels on the horizontal electrophoresis unit due to their fragility. The gel dimension was 8 × 8 cm. 4 μL of samples were manually loaded into the wells formed in the gels using a micropipette.

### 2.3. Electrodes Design

The clamp-shaped electrodes were manufactured by folding platinum-coated titanium plates twice. Both gel ends were covered with wet paper wicks and inserted into the clamp-shaped electrodes. For the double-deck flat electrodes, two pairs of platinum-coated titanium strips were placed at the top and bottom of the gels. The wet paper wicks were cut to the shape of the electrodes and inserted between the gels and electrodes.

### 2.4. FIGE Module

A schematic representation of the pulsing circuitry is provided in Supplementary Figure S2. A picture of the prototype FIGE module used in gel electrophoresis can be found in Supplementary Figure S3. Forward and reverse switching of the electric field supply to the gel was achieved by interfacing the voltage supply (PS300HC, BIOFACT, Daejeon, Republic of Korea) with a 400 V direct current relay (G9EJ-1-P-E, OMRON, Kyoto, Japan) that could handle a current of 15 Amp. The forward and reverse switching rate was controlled using an Arduino Uno microcontroller that could deliver pulses as short as one msec. The performance of this instrumentation regarding square waveform, amplitude, length, and stability was checked and ascertained using an oscilloscope (DSO3152A, Agilent Technologies, Santa Clara, CA, USA).

### 2.5. Horizontal SDS-PAGE

Flatbed Professional, a horizontal flatbed chamber (FC-EDCProf-2836, Gel Company, USA), was used as the platform for all electrophoretic separations. Electrophoresis was performed with either its original platinum wire electrodes, the clamp-shaped electrodes, or the double-deck electrodes. The distance between an anode and a cathode electrode was 6 cm. The temperature of the cooling plate was set to 6 °C. The running buffer was prepared using a 25 mM tris base, 192 mM glycine, and 0.1% SDS with a pH of 8.3. Also, 5 μL of pre-stained protein marker was loaded into four sample wells (2 × 2 × 2 mm) and separated under a constant current of 50 mA for 90 min. The voltage was usually started around 70 V and gradually increased to 120 V. The bromophenol blue dye traveled for about 3 cm. The temperature changes during the electrophoretic separations were measured with three digital thermometers with a K-type thermocouple probe. The thermometer probes were inserted into the gel’s top, bottom, and middle portions.

Pulsed field electrophoresis was achieved by interfacing a power supply with the FIGE module. A constant current of 50 mA was applied with an alternating forward pulsing time of 3 sec and reverse pulsing time of 1 sec. Separation continued until the bromophenol blue dye had traveled about 3 cm.

After electrophoresis, an image of the gel was scanned using an optical scanner (SL-C486FW, Samsung, Suwon-si, Republic of Korea). The gel was also longitudinally cleaved along each lane, and the cross-sectional images were obtained using an optical scanner.

## 3. Results

We explored the less frequently discussed topic of the electrode design in horizontal PAGE systems and its effects on the gel resolution. In typical vertical PAGE systems, two electrodes are submerged into separate running buffer reservoirs. An electric field is applied using the running buffer and enters the gel from the ends in the migrating direction. This configuration allows the uniform distribution of electrophoretic force inside the gels. For horizontal PAGE, however, two long electrodes are positioned on top of the gel at each end. Multiple layers of paper wicks are wetted with the running buffer and placed as interfaces.

The standard electrodes for the horizontal system were made using thin platinum wire, also tested in this study. Then, variation of the electrode was introduced by using a thicker titanium rod with a conductive polymer cover that provided better conductivity (Serva Electrophoresis, Heidelberg, Germany). To apply a uniform electric field to the gel, thin metal tapes were cut to the cross-sectional size of the gel, attached to the migration ends, and used as electrodes. This configuration showed some promising upright protein band patterns but was impractical to use. Because it was thin and fragile, the gel was easily wrinkled or even torn when placing the electrodes. It was also challenging to maintain proper contact with thin gel during the operation.

**Figure 1:**
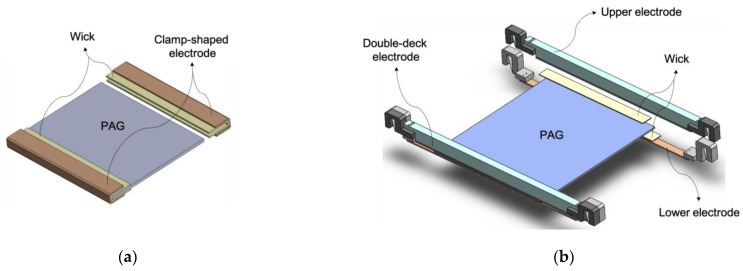

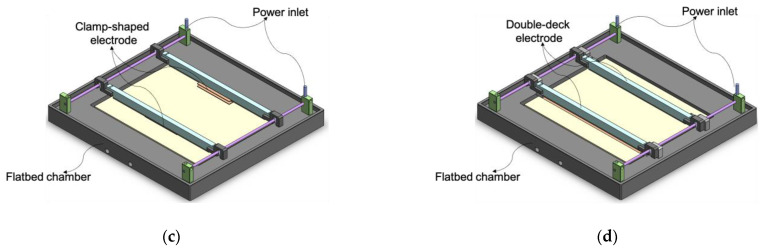
The perspective views of the clamp-shaped electrode and double-deck electrode. Schematic images of the clamp-shaped electrode (**a**) and the double-deck electrode (**b**). Both electrodes were installed in a flatbed electrophoresis unit (**c**,**d**).

Two new electrodes were designed for an even electric field during electrophoresis and easier installation. The clamp-shaped electrode (Figure 1a) was manufactured by folding a platinum-coated titanium plate twice into a Π shape from the side. Each end of the gel was wrapped in running buffer-soaked paper wicks and slid into the electrodes. This electrode provided an electric field from the gel’s top, bottom, and side. However, the insertion of the gel into this electrode was still cumbersome. The double-deck electrode (Figure 1b) was developed to ease the handling difficulty. It consists of two sets of platinum-coated titanium strip pairs that sandwich the gel from the top and bottom. Since each strip pair was connected to the same power outlet, the same electric fields were applied simultaneously to the top and bottom of the gel. The new electrodes were tested in a flatbed electrophoresis unit, replacing the original wire electrode (Figure 1c,d).

**Figure 2:**
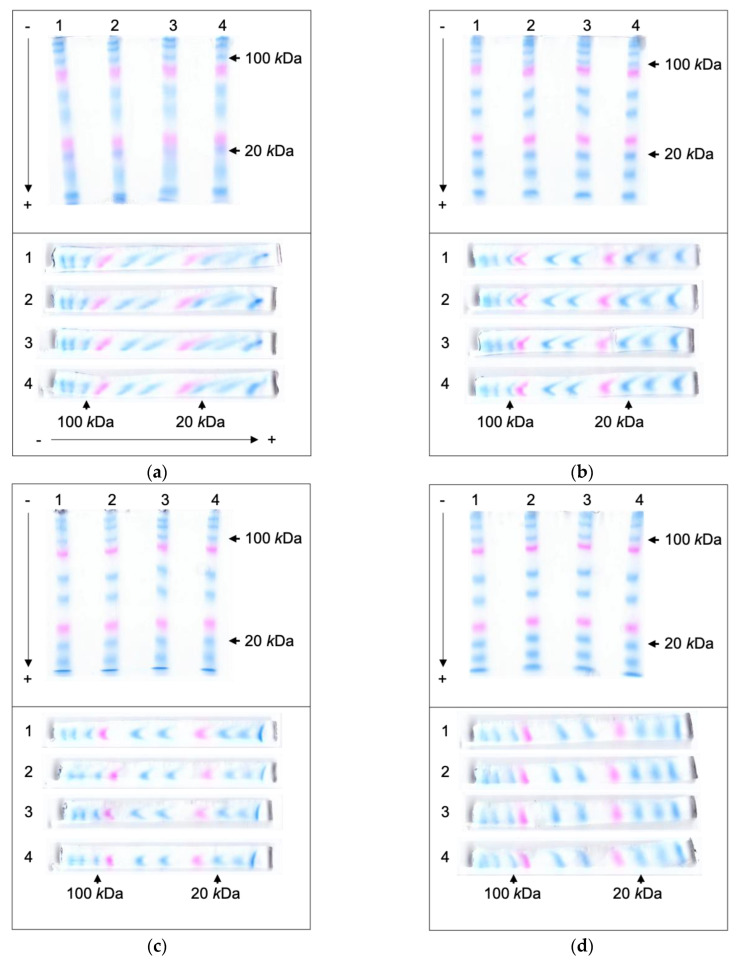

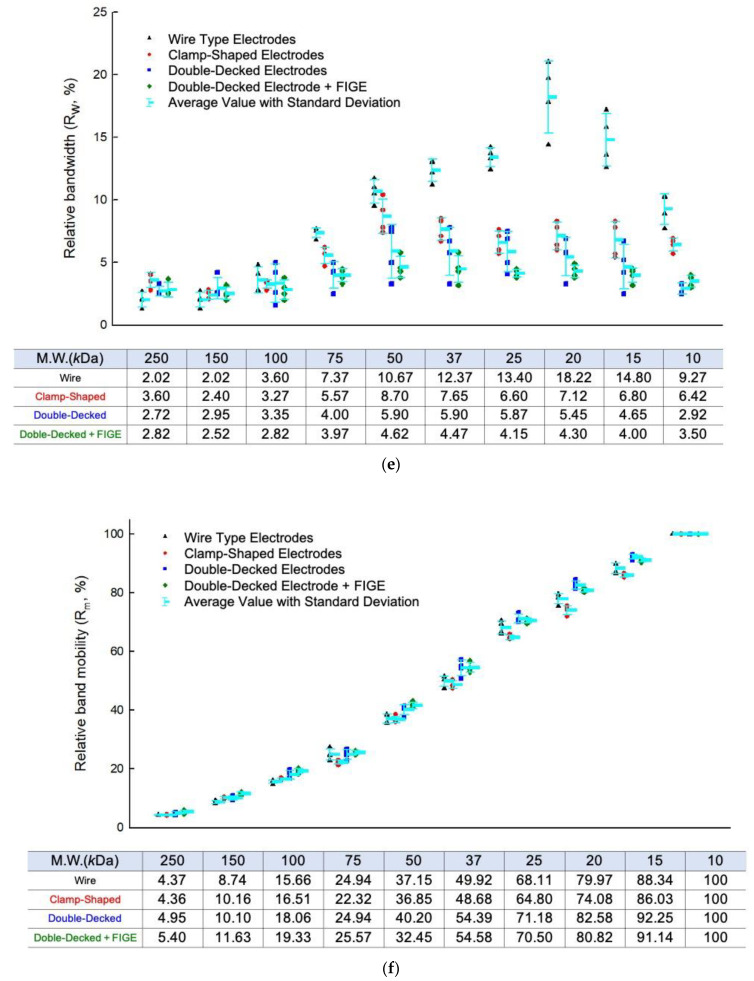
Electrophoretic separation of pre-labeled marker proteins in horizontal PAGE. Electrophoresis was performed in a flatbed chamber with the genuine wire electrodes (**a**), the clamp-shaped electrodes (**b**), the double-deck electrodes (**c**), and the double-deck electrodes in conjunction with the FIGE module (**d**). Precision Plus Protein™ Dual Color Standards pre-labeled markers (Bio-Rad) were used to monitor the movement of proteins in gel. Estimated molecular weights of marker proteins were 250, 150, 100, 75 (red), 50, 37, 25 (red), 20, 15, 10 kDa. The location of 100 and 20 kDa proteins were marked within the images. After electrophoretic separations, whole gels were scanned (upper panel) and longitudinally cleaved along each lane. The cross-sectional images of four sample lanes were also obtained using an optical scanner (lower panel). The directions of electrophoresis are shown with arrow lines. The relative bandwidths (R_w_) and the relative band mobilities (R_m_) were determined from the gel images (**e**,**f**).

The pre-stained size markers were separated in 10% tris-glycine polyacrylamide gels with a flatbed electrophoresis unit to evaluate the protein migration patterns in horizontal PAGE. This electrophoresis instrument design has been used for over 40 years without significant modifications [4]. Platinum wire electrodes were placed on the top of the gel at both ends in the migrating direction. After electrophoretic separation, the shapes of the protein bands were monitored from the top and the lateral side of a gel (Figure 2a). The protein mobility and the protein separation efficiency (i.e., relative bandwidth in the direction of separation) were determined from the gel images (Figure 2e,f). The relative band mobility (R_m_) was defined as the percentile ratio of the target protein’s migration distance to the dye front’s migration distance. Similarly, the relative bandwidth (R_w_) was defined as the percentile ratio of the target protein’s bandwidth to the dye front’s migration distance.

In conventional electrophoresis using wire electrodes, the electric field strength weakens from the top to the bottom, resulting in uneven protein migration. After electrophoretic separation, lateral views of the gel revealed that proteins in the upper section of the gel migrated a greater distance, leading to protein band shapes that slanted toward the anode (Figure 2a). This phenomenon was due to the relatively stronger electric field at the top of the gel, which accelerated protein movement. The difference in protein migration speed between the upper and lower parts of the gel accumulated as the proteins moved, thus causing this effect to be particularly noticeable for proteins with molecular weights lower than 100 kDa, as they migrated a greater distance during electrophoresis. The slanting band shapes caused band broadening in the top view and impeded the differentiation between individual protein bands. The broadening of bands was most prominent with the 20 kDa marker protein, with an R_w_ of 18.2.

The protein bands were more focused with these new electrodes and showed better distinctions between them (Figure 2b,c,e,f). The side views of the gels showed that all bands were thinner and more focused. For all the proteins with molecular weights lower than 100 kDa, which were skewed a lot with a wire electrode, the R_w_ values were significantly lower. The R_w_ of the 20 kDa marker protein was improved to 7.1 and 5.5 with the clamp-shaped and the double-deck electrodes, respectively. However, the R_m_ of the bands was similar regardless of the electrode type.

As the protein bands migrated farther in the gel, they started to form a crescent shape with their ends pointing toward the anode. This tendency was more prominent with the double-deck electrodes. It could be assumed that the electric fields were stronger and caused faster migration at the gel’s top and bottom. Such deformation also caused band broadening. Therefore, the FIGE technique was adopted to remove these pointy trails and to focus the protein bands for enhanced separation (Figure 2d–f). The FIGE module interfaced between a power supply and the double-deck electrodes. Since the FIGE module periodically reversed the direction of the electric field, it took longer for the protein bands to migrate for a similar distance as before. All the bands were thinner and aligned nearly vertically for the best resolution. The R_w_ of the 20 kDa marker protein was further decreased down to 4.3.

During electrophoresis, the gels had to be cooled down to actively remove heat and minimize the evaporation of the running buffer. Joule heating can affect the electrophoretic mobility of proteins, leading to band broadening [12]. To monitor the spatial temperature differences, K-type thermocouple wire probes were inserted into the gel’s top, middle, and bottom sections, and the temperatures were measured during electrophoresis. The temperature at the bottom of the gels was set to 6 °C with the cooling plate. Regardless of the electrode types, the temperatures were slightly higher at the top, but the differences were smaller than 2 °C. The measurements remained similar for up to 2 h.

## 4. Discussion

This study investigated a long-neglected fault in the electrode design in the horizontal protein PAGE system. The method of applying an electric field in a gel was a critical factor for better resolution. Two new electrodes were developed and proven useful to get narrow and focused protein bands. With the expenditure of a longer electrophoresis time, the FIGE technique could be used together with new electrodes to get nearly vertical protein band shapes.

In side-by-side comparisons, both the clamp-shaped and double-deck electrodes showed enhanced protein separations similarly. Even for the clamp-shaped electrode, like the double-deck electrode, the major contact with the gel was made at the gel’s top and bottom. The input from the end should be marginal. It can be assumed that the electric field was applied similarly with both electrodes. Therefore, because of the ease of its installation, the double-deck electrode is recommended for laboratory use.

The depicted two electrodes followed the size, electric connection, and other specifications of the Flatbed Professional horizontal flatbed chamber unit (FC-EDCProf-2836) from Gel Company in particular. However, when used with other horizontal electrophoresis systems, appropriate changes could be made without departing from the principles and spirit of these embodiments.

The original intention was that two electrodes would sandwich the gel from the same location. As shown in Figure 2d as an example, the gels might still have bands slightly leaned forward or even backward. These variations were observed to differ slightly when different types of gels, with varying thickness and acrylamide concentrations, were tested with the new electrodes. Additionally, differences in the Joule heat generation and ambient humidity during the electrophoretic operations could influence the dryness of the gel and subsequently lead to these variations. As a remedy, the strength of the electric field at the top and the bottom of the gel might be independently adjusted. When the distances between the two electrodes in the gel narrowed, the electric currents were measured to have increased (Supplementary Figure S4). Therefore, the electric field could be easily fine-tuned by adjusting the interval between the electrodes at the top or the bottom of the gel. For the flatbed electrophoresis unit used in this study, the electrodes were electrically connected via guiding rails on the side. The positions of electrodes could be easily changed by sliding them along the guiding rails.

Two new types of electrodes were introduced for horizontal electrophoresis, which can be easily manufactured and installed in most commercially available systems without the need for significant hardware modifications. Additionally, they are user-friendly. Various other techniques and products, such as different electrophoresis buffers, pre-cast gels, improved electrophoresis devices, and diverse electrophoresis techniques, exist to enhance the quality of electrophoresis. Given the distinct advantages of these new electrodes over other technologies, they have the potential to effectively enhance the quality of horizontal electrophoresis when used in combination with these methods.

## Data Availability

No additional data were created or analyzed in this study. Data sharing is not applicable to this article. The authors can be contacted for any further information regarding the data within the article.

## Supplementary Materials

The following supporting information can be downloaded at https://www.mdpi.com/article/10.3390/mps6060106/s1. Figure S1: Polyacrylamide gel caster; Figure S2: Electric circuit of the pulse generator in the FIGE module; Figure S3: The physical setup of the horizontal PAGE system with the FIGE module; Figure S4: The intervals between the electrodes could adjust electric currents.
