# Supplementary material for: Improved Separation in Horizontal Protein SDS-PAGE with Double-Deck Flat Electrodes and a Field Inversion Gel Electrophoresis Module
Source: Methods Protoc. 2023 Nov 3;6(6):106. doi: 10.3390/mps6060106 (PMC10660703; doi:10.3390/mps6060106)
Supplement: Supplementary file 1 [file mps-06-00106-s001.zip › mps-2653985-supplementary.pdf]

Supplementary Material

Improved separation in Horizontal protein SDS-PAGE with Double-deck flat electrodes and Field-inversion gel electrophoresis module

Dong Woo Lim[1,‡], Tae-Sung Yoon[2,‡], Kyung Ho Han[3], Saba Sajjad[2], Heung-Seon Shin[1], Sunghyun Kang[1,2,*]

[1] T-MAC Co. Ltd., Yuseong-gu, Daejeon 34141, Korea
[2] Critical Diseases Diagnostics Convergence Research Center, Korea Research Institute of Bioscience and Biotechnology (KRIBB), Yuseong-gu, Daejeon 34141, Korea
[3] Department of Biological Sciences and Biotechnology, Hannam University, Yuseong-gu, Daejeon 34054, Korea

‡ Equal contributors
*Correspondence should be addressed to the following author:
Full name: Sunghyun Kang Ph.D.
Department: Critical Diseases Diagnostics Convergence Research Center
Institution: KRIBB
Address: 125 Gwahak-ro, Yuseong-gu, Daejeon 34141, Korea
Email: skang@kribb.re.kr

Supplementary Figure S1

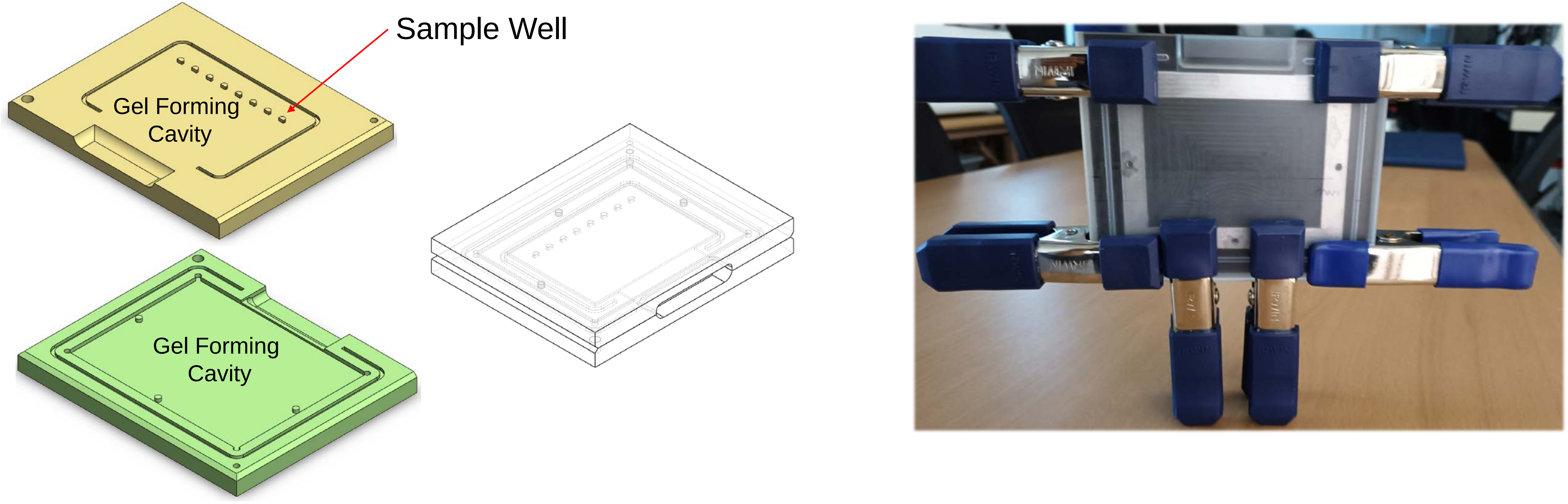

(a) (b)

Figure S1 Polyacrylamide gel caster. Perspective views of the polyacrylamide gel caster parts (a). After the assembly of the caster, the polyacrylamide gel mixture was poured into the cavity of the caster and polymerized (b).

Supplementary Figure S2

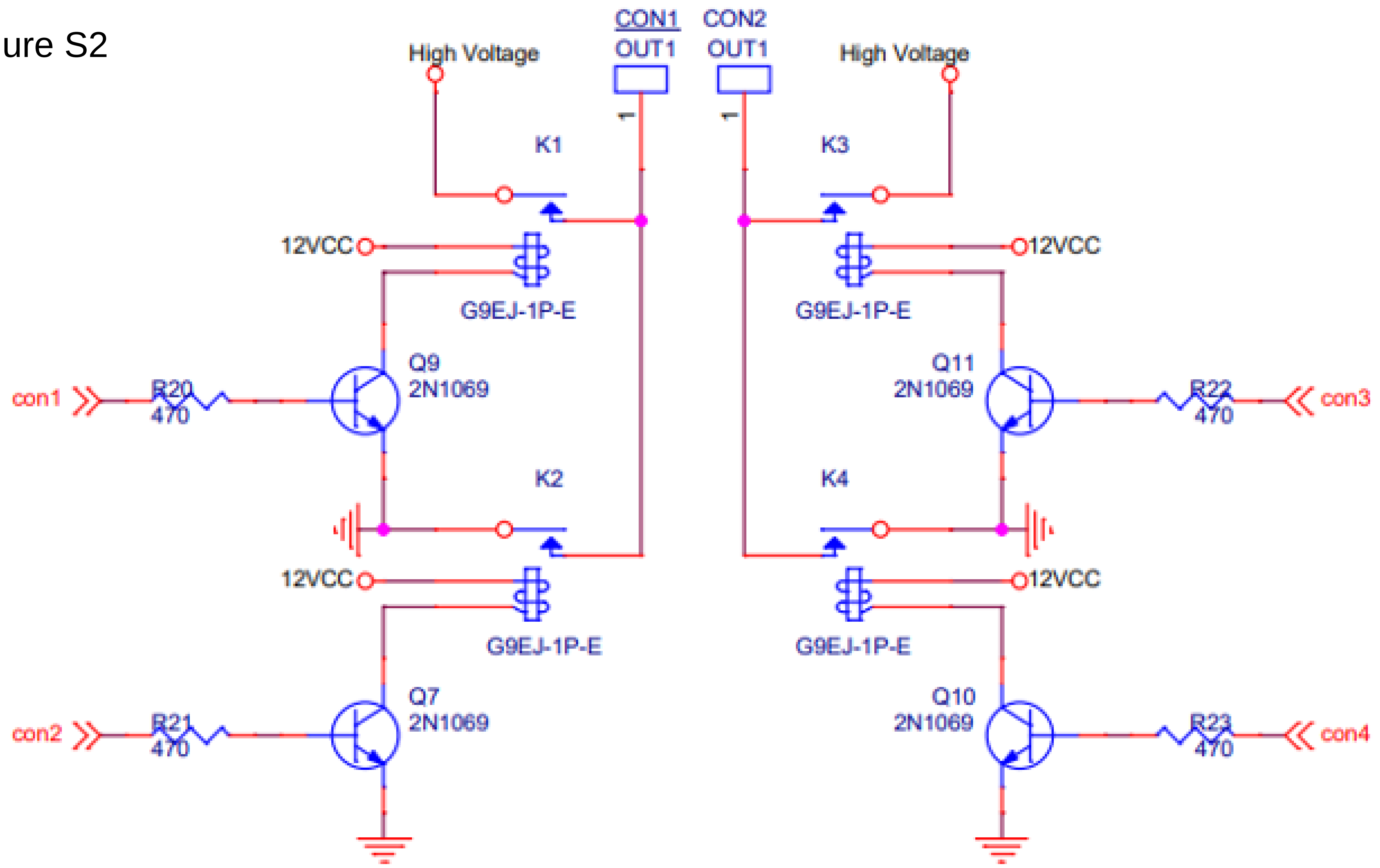

Figure S2 Electric circuit of the pulse generator in the FIGE module. The electric circuit generated positive and negative square-wave electric fields during field inversion experiments.

Supplementary Figure S3

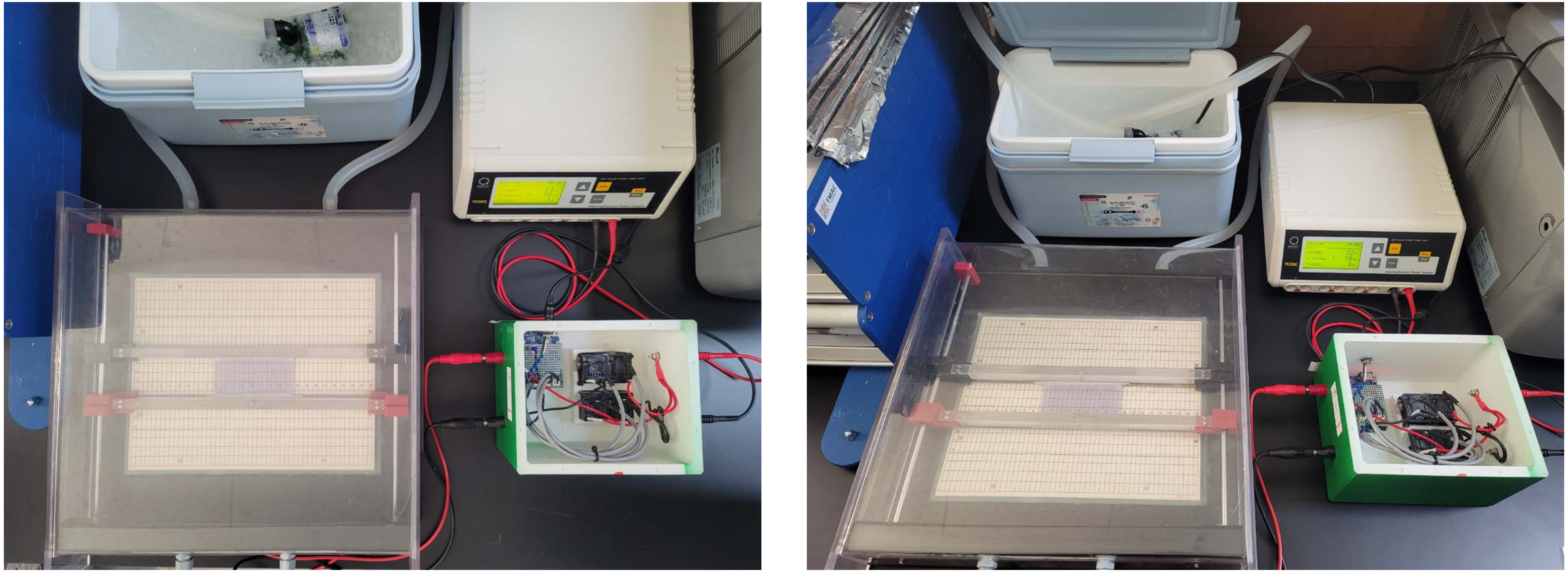

Figure S3 The physical setup of the horizontal PAGE system with the FIGE module. The FIGE module linked the power pack to the horizontal electrophoresis device.

Supplementary Figure S4

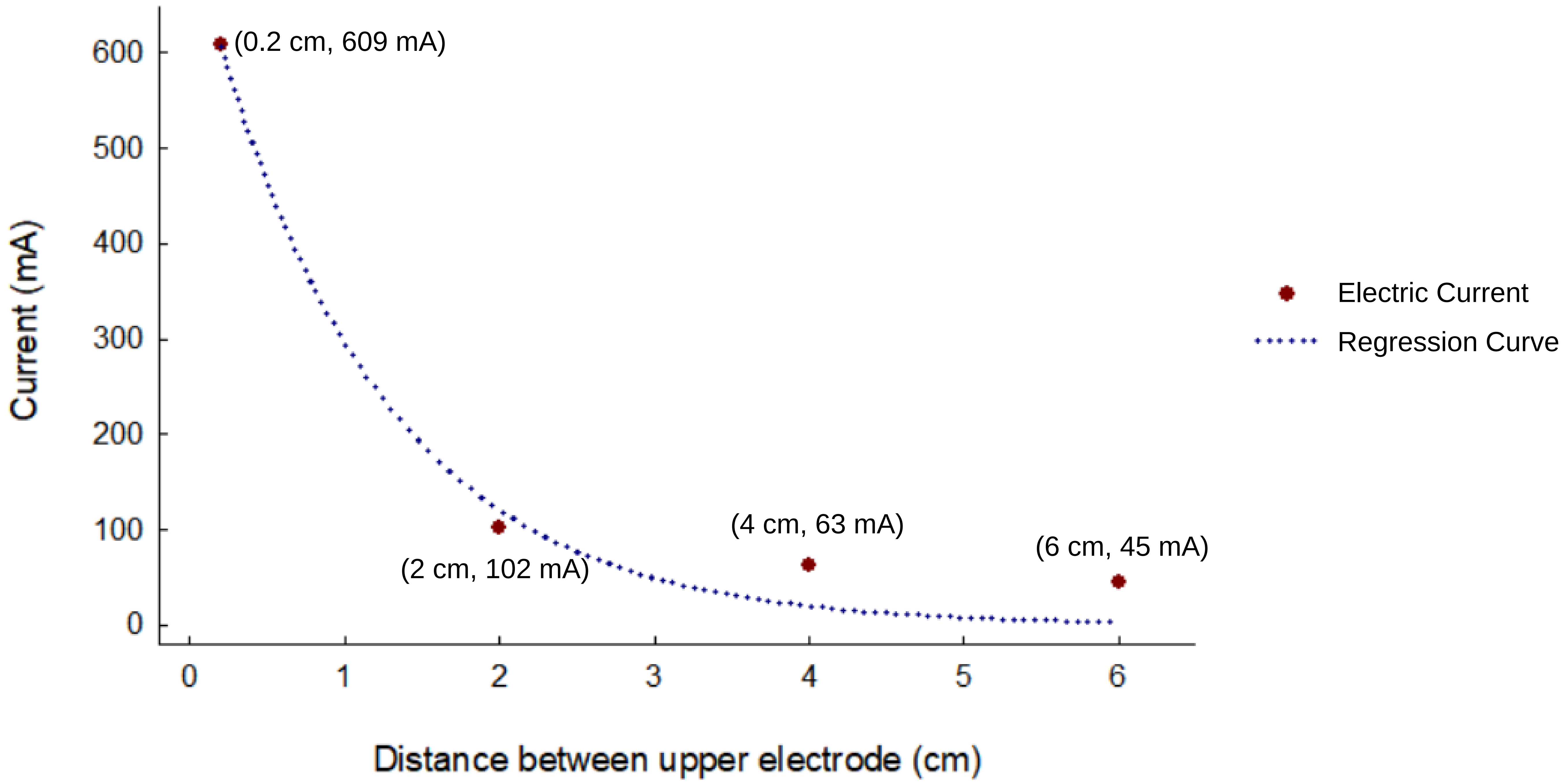

Figure S4 The intervals between the electrodes could adjust electric currents. Two upper electrodes from the double-deck electrode module were placed on the top of a gel with varying intervals, and an electric field was applied (50 V). As the intervals narrowed, the electric currents were measured to increase exponentially. The data was fitted with an exponential decay curve (y = 726.2*exp(-0.897*x), r2 = 0.982).
